# Acromegaly, Herniation of Cerebellar Tonsils, and Arnold–Chiari 1 Malformation: The Importance of Right Definitions

**DOI:** 10.1155/2024/4733399

**Published:** 2024-03-20

**Authors:** Alessandro Ciarloni, Gianmaria Salvio

**Affiliations:** Endocrinology Clinic, Department of Clinical and Molecular Sciences, Polytechnic University of Marche, Ancona 60126, Italy

## Abstract

We present a case of acromegaly associated with Arnold–Chiari 1 malformation and a literature review regarding this association, mainly focusing on the importance of a clear distinction between Chiari malformation and herniation of cerebellar tonsils (CTH). Indeed, in many clinical cases, this distinction has not been properly made and a better description of the radiological findings could be important for the clinical management of these patients. In fact, Arnold–Chiari 1 malformation, as a congenital disease, is not caused by acquired growth hormone (GH) excess, but the latter could worsen pre-existing CTH or even induce it *ex novo*. Therefore, awareness of this condition in the clinical management of acromegaly appears crucial.

## 1. Introduction

The Arnold–Chiari malformation is a congenital underdevelopment of the posterior cranial fossa that may lead to the displacement of the cerebellar tonsils in the magnum foramen, and it is divided into four types, with type 1 being the one with the minor protrusion of the cerebellar tonsils and often asymptomatic and the type 4 being the one with the major protrusion and more significant symptoms [1].

There is a well-known association between Arnold–Chiari malformation and other intracranial abnormalities such as Dandy–Walker cyst, congenital hydrocephalus, holoprosencephaly, polymicrogyria, and chromosomal anomalies such as trisomy 18, 13, and 8 and midline development abnormalities, such as agenesis of the corpus callosum, the latter being often associated with pituitary hormone deficiencies due to congenital alterations of the pituitary transcription factors [2].

There is also an association between acromegaly (a rare endocrine disease caused by growth hormone (GH) excess) and skull deformations such as skull base fibrous dysplasia, especially in the context of McCune–Albright syndrome that is characterized by poly/monostotic fibrous dysplasia, café-au-lait spots, and hyperfunctioning endocrinopathies [3]. Fibrous dysplasia seems also to be associated with cranial base abnormalities such as Arnold–Chiari malformation type 1, probably due to cranial constriction and cranial settling likely resulting from increased pression of craniofacial lesions on a weak skull base. In patients with McCune–Albright syndrome, there are also potentially treatable factors that may be involved in the development of the malformations, such as FGF23-mediated hypophosphatemia, hyperthyroidism, precocious puberty, and scoliosis [4].

The association between Chiari malformation and acromegaly has been described in some previous papers but never deeply understood [5–12]. We describe here a case of a patient with the association of Arnold–Chiari malformation type 1 and acromegaly, and we propose a literature review about this association.

## 2. The Case

The patient was a 61-year-old woman who had a history of psoriasis localized in the ear area, in the head, on the forearm, on the hands, and on the foot fingers, associated with desquamative erythema. For this reason, she was in treatment with calcipotriol, betamethasone, and ixekizumab.

Other anamnestic elements of interest were as follows: ureterolithotripsy with the introduction of ureteral stent JJ for kidney stones, hysterectomy, and monolateral left ovariectomy due to uterine fibromatosis, cholecystectomy due to cholelithiasis, mammarian right nodulectomy, surgically treated rectocele, and rectal prolapse.

Due to the dermatological problem, the patient was hospitalized in the Dermatological Clinic of the university hospital “Azienda Ospedaliera Universitaria delle Marche” in December 2022, where due to hypercholesterolemia and diabetes she underwent an endocrinological evaluation. Since characteristic facies with macroglossia and prognathism, history of snoring and enlargement of hands and feet raised the clinical suspicion of acromegaly, and a screening test was performed, revealing significantly high serum insulin-like growth factor 1 (IGF-1) levels (410 mcg/l–normal values for age and gender 15–190 mcg/l).

Subsequently, the patient was hospitalized in the Clinic of Endocrinology of the university hospital “Azienda Ospedaliera Universitaria delle Marche” in February 2023. The diagnosis of acromegaly was confirmed by repeated serum IGF-1 measurement (416 mcg/l–normal values for age and gender 15–190 mcg/l) and lack of suppression of the serum GH below 0.4 ng/ml after a 75 oral glucose load (Table 1).

**Figure 1:**
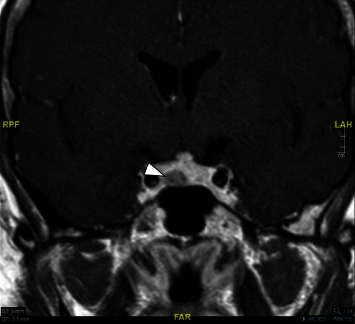
Expansive adenomatous lesion in the sellar region.

No other pituitary hormone deficits have been noticed at the biochemical evaluations. Brain magnetic resonance imaging (MRI) was then performed, confirming the presence of an expansive lesion in the sellar region and describing it as adenomatous, measuring 0.84 × 0.55 cm (Figure 1).

In order to study possible complications of acromegaly, an ultrasound evaluation of the thyroid gland revealing a multinodular goiter (with normal thyroid function confirmed by blood tests) and a colonoscopy, with the founding of multiple polyps (that were of hyperplastic nature at the histological evaluation) were performed.

An evaluation of the calcium-phosphorus metabolism has been performed with the discovery of elevated levels of bone alkaline phosphatase, so a bone scintigraphy was performed, and a hypercaptation in the sternal manubrium, in the skull case, and in the cervical spine was highlighted. These findings were interpreted as psoriatic arthritis, and a targeted radiological examination did not highlight focal alterations, so a subsequent dermatological revaluation was suggested.

The patient underwent medical treatment, starting with short-acting octreotide and later with long-acting octreotide (lanreotide 90 mg one subcutaneous injection every 28 days).

**Figure 2:**
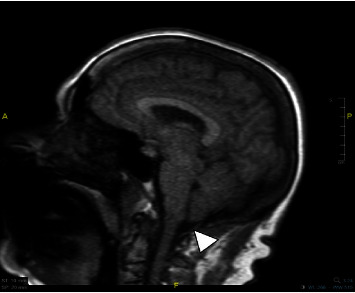
Displacement of the cerebellar tonsils in the magnum foramen.

Interestingly, the patient had also a history of Arnold–Chiari malformation type 1 without signs of myelopathy or syringomyelia (Figure 2), discovered for the first time in June 2018, which was stationary at a brain MRI in January 2020. The Chiari 1 malformation was asymptomatic. Notably, during the latter exam, a globular pituitary gland was discovered with the association of an espansive oversaddle median-paramedian right lesion of 1.2 cm of nonclear interpretation. In that MRI, the Arnold–Chiari malformation type 1 was defined as stable.

At a following endocrinological evaluation in May 2023, the acromegaly was in good biochemical (IGF-1 levels 141 mcg/l–normal values for age and gender 15–190 mcg/l) and clinical control with the therapy described before, so it was confirmed.

In a subsequent endocrinological evaluation of July 2023, despite the persistent good biochemical control (IGF-1 levels 130 mcg/l–normal values for age and gender 15–190 mcg/l), the clinical features characterized by enlargement of the hands, tongue, and feet led to optimization of the medical therapy (lanreotide 90 mg one subcutaneous injection every 28 days). In that evaluation, due to newly referred postural instability and history of Arnold–Chiari 1 malformation, a neurological evaluation was suggested.

## 3. Conclusions

The association between Chiari 1 malformation and acromegaly has been described in some other reports [5–12], and a possible cause-effect relationship has been hypothesized. Indeed, the effect of GH on brain volume or to the bone hypertrophy related to GH excess may lead to cranial constriction and higher intracranial pressure, resulting in a predisposition to herniation of cerebral tonsils (CTH). However, a clear distinction between Chiari malformation and CTH should be made. CTH is defined as a herniation of more than 3 mm through the foramen magnum of the cerebellar tonsils, while the Chiari malformation is the specific condition where this herniation is related to the congenital underdevelopment of the posterior cranial fossa. In fact, CTH could also be acquired, i.e., in cases of hydrocephalus or intracranial masses that cause an increased intracranial volume [13]. The main feature of Chiari 1 malformation is usually a shorter clivus, while in acquired CTH, posterior cranial fossa changes are not expected. In a study on 150 patients with acromegaly, the incidence of CTH on MRI evaluation was significantly higher than in a control group of subjects who underwent MRI evaluation for headache or transient neurological deficits. In that study, a second control group was composed of 24 symptomatic classic Chiari 1 malformation patients, and in those patients, the clivus length was significantly shorter than that in the acromegaly patients with CTH, suggesting that acromegaly could cause an acquired form of CTH but not structural anatomical alteration of the posterior cranial fossa [9].

In a few cases, the neurosurgical treatment of GH-secreting pituitary adenoma has led to the reduction of a concomitant CTH [6, 10]. In one of those case reports, a patient with GH-secreting pituitary adenoma and CTH associated with cervical syringomyelia showed a complete resolution of CTH at an MRI evaluation performed 10 months after the neurosurgical treatment (transsphenoidal resection) of acromegaly with subsequent sellar irradiation [10]. In that study, the anatomical structure of the posterior cranial fossa has not been described, and only the CTH has been evaluated, so the presence of a Chiari 1 malformation was not clearly described. Of note, CTH and syringomyelia seemed reversible after acromegaly resolution.

In another study, a case of GH-secreting pituitary adenoma and Chiari 1 malformation, with small posterior fossa and CTH, was described. In that patient, the Chiari malformation was associated with syringomyelia and thoracic spine scoliosis. In the MRI evaluation, the pituitary adenoma had a suprasellar extension with destruction of sella turcica. After surgical treatment, normalization of GH and IGF-1 levels was reached, and at the postsurgical MRI evaluation, the adenoma was not detectable [8]. In this paper, features regarding the Chiari 1 malformation in the postsurgical time are not described, so the evolution of the pathology in correlation with the normalization of GH and IGF-1 levels was not evaluated.

In the other two reported cases [5, 7], the Chiari 1 malformation was associated with syringomyelia and important compression of the medulla that required decompression of the foramen magnum before the treatment of a concomitant acromegaly. In the first case, GH-secreting pituitary adenoma was treated by neurosurgery followed by radiation therapy and then subcutaneous octreotide [7], whereas in the second case, only medical therapy with long-acting somatostatin analog was chosen due to concomitant colon cancer that required surgery and chemotherapy [5]. In these cases, it was impossible to evaluate the correlation between acromegaly and Chiari malformation, but it should be noted that acromegaly seems also associated with cases of severe symptomatic Chiari malformation.

In our patient, the CTH was described at the MRI evaluation of 2018 and the pituitary adenoma was described for the first time in 2020. For this reason, the cause-effect relationship between acromegaly and CTH does not seem probable. However, in consideration of case reports previously described [6, 10], it would be important to evaluate the evolution of the CTH in the next MRI evaluation considering the normalization of GH and IGF-1 levels after the introduction of the medical treatment. On the other hand, even if a cause-effect relationship between acromegaly and Chiari 1 malformation has not yet been demonstrated, GH excess seems to be associated with an increased risk of CTH [9]. For this reason, CTH seems to be an important parameter to properly consider during the clinical, biochemical, and instrumental follow-up of our patient. The concomitant onset of postural instability and enlargement of hands, tongue, and feet despite the good biochemical control of acromegaly is another aspect to consider and evaluate to better understand the complex association of those two diseases, since it may suggest that the biochemical evaluations could be important but not enough to a complete evaluation of the problem. In this context, a worsening of CTH-related symptoms could be an additional factor to evaluate while considering neurosurgical treatment of the GH-secreting pituitary adenoma, especially considering that neurosurgery may also lead to the reduction of CTH [6, 10].

Finally, we had the diagnosis of Chiari 1 malformation, but the developmental status of the posterior cranial fossa has not been described in the MRI evaluations so it would be important to better describe the anatomy of the posterior cranial fossa in the next MRI evaluation to understand the nature of the CTH and the association with acromegaly. This description would be important also in the case of a future need of a neurosurgical approach because the management of Chiari 1 malformation and CTH is different [1]. A better morphological description of the skull base to evaluate the presence of fibrous dysplasia could also be important, because that alteration is known to be associated with Chiari 1 malformation and McCune–Albright syndrome as well, which in that case should be ruled out [3, 4].

## Figures and Tables

**Table 1:** Oral glucose load test.

	Timepoint (minutes)				
	0	30	60	90	120
GH (ng/ml)	1.82	1.47	3.26	3.64	2.64
Glucose (nmol/L)	5.99	9.16	12.77	15.26	11.93

GH = growth hormone.

## Data Availability

The data used to support the findings of the study are available from the corresponding author upon request.
